# Ischemic A/D transition of mitochondrial complex I and its role in ROS generation^☆^

**DOI:** 10.1016/j.bbabio.2015.12.013

**Published:** 2016-07

**Authors:** Stefan Dröse, Anna Stepanova, Alexander Galkin

**Affiliations:** a Clinic of Anesthesiology, Intensive-Care Medicine and Pain Therapy, University Hospital Frankfurt, Frankfurt am Main 60590, Germany; b Medical Biology Centre, School of Biological Sciences, Queens University Belfast, 97 Lisburn Road, Belfast BT9 7BL, UK; c Feil Family Brain and Mind Research Institute, Weill Cornell Medical College, 407 East 61st Street, New York, NY 10065, USA

**Keywords:** A/D, active/deactive transition, DQA, 2-n-decyl-quinazolin-4-yl-amine, EPR, electron paramagnetic resonance, FMN, flavin mononucleotide, IMS, intermembrane space, I/R, ischemia/reperfusion, NAD^+^/NADH, oxidized/reduced form of nicotineamide adenine dinucleotide, NEM, *N-*ethylmaleimide, NO, nitric oxide, OGDH, 2-oxoglutarate dehydrogenase, PDH, pyruvate dehydrogenase, Q, ubiquinone, RET, reverse electron transfer, RNS, reactive nitrogen species, ROS, reactive oxygen species, SMP, submitochondrial particles, Mitochondrial complex I, Ischemia/reperfusion injury, ROS generation, A/D transition, Thiol redox modification

## Abstract

Mitochondrial complex I (NADH:ubiquinone oxidoreductase) is a key enzyme in cellular energy metabolism and provides approximately 40% of the proton-motive force that is utilized during mitochondrial ATP production. The dysregulation of complex I function – either genetically, pharmacologically, or metabolically induced – has severe pathophysiological consequences that often involve an imbalance in the production of reactive oxygen species (ROS). Slow transition of the active (A) enzyme to the deactive, dormant (D) form takes place during ischemia in metabolically active organs such as the heart and brain. The reactivation of complex I occurs upon reoxygenation of ischemic tissue, a process that is usually accompanied by an increase in cellular ROS production. Complex I in the D-form serves as a protective mechanism preventing the oxidative burst upon reperfusion. Conversely, however, the D-form is more vulnerable to oxidative/nitrosative damage. Understanding the so-called active/deactive (A/D) transition may contribute to the development of new therapeutic interventions for conditions like stroke, cardiac infarction, and other ischemia-associated pathologies. In this review, we summarize current knowledge on the mechanism of A/D transition of mitochondrial complex I considering recently available structural data and site-specific labeling experiments. In addition, this review discusses in detail the impact of the A/D transition on ROS production by complex I and the S-nitrosation of a critical cysteine residue of subunit ND3 as a strategy to prevent oxidative damage and tissue damage during ischemia–reperfusion injury. This article is part of a Special Issue entitled Respiratory complex I, edited by Volker Zickermann and Ulrich Brandt.

## Highlights

• The current knowledge on active/deactive (A/D) transition of complex I is reviewed.
• The mechanism and driving force of the A/D conformational change are discussed.
• The A/D transition can affect ROS production and ischemia/reperfusion injury.

## Graphical abstract

**Figure:**
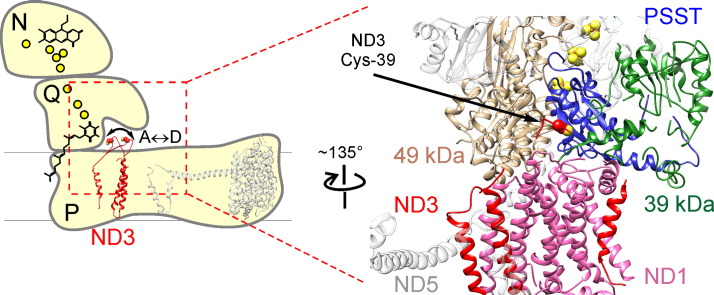

## 1 Introduction

The aerobic catabolism of carbohydrates, lipids, and proteins by eukaryotes, which provides energy for cellular needs, includes the transfer of electrons originating from metabolites to NAD^+^, the universal carrier of reducing equivalents*.* Complex I (NADH:ubiquinone oxidoreductase, Type I NADH dehydrogenase) of the mitochondrial respiratory chain catalyzes NADH oxidation by regenerating NAD^+^. This giant enzyme is located in the inner mitochondrial membrane and remarkable recent progress in understanding its molecular structure [1], [2], [3] is reviewed in this special issue (see especially the articles of Zickermann, Sazanov, and Brandt). Since the mammalian enzyme is a large complex with 7 out of 44 subunits encoded in mitochondrial DNA (i.e., the ND subunits), genetic defects in the oxidative phosphorylation system can originate from mutations in either nuclear or mitochondrially encoded subunits of complex I. Complex I defects can alter energy metabolism and are linked to multisystemic disorders manifested in early childhood in highly metabolizing tissues like brain and heart [4].

During NADH oxidation by complex I (forward reaction), electrons are transferred from the primary electron acceptor FMN via a chain of FeS-clusters to ubiquinone, the hydrophobic electron carrier in the inner mitochondrial membrane. The free energy change of this redox reaction drives the translocation of four protons across the membrane [5], [6], [7], contributing 40% to the formation of the proton-motive force that is utilized by ATP-synthase for the production of ATP. Complex I holds a key role in energy metabolism as the main consumer of NADH in the mitochondrial matrix.

Since electron transfer from NADH to ubiquinone and proton translocation are spatially separated, conformational change-driven models of coupling are the consensus in the field [1], [8], [9], [10]. At least two different semiquinone intermediate signals were identified in complex I by EPR [11], [12], and therefore most of the proposed mechanisms include a conformational change driven by production [3] or stabilization (so-called E and P-states) [8] of negatively charged semiquinone molecules. However, the exact coupling mechanism of energy transduction for complex I is still not resolved.

The catalytic properties of eukaryotic complex I are profoundly multi-facetted (see [13] for a review). The reaction catalyzed by complex I is fully reversible, and at the expense of proton-motive force, the enzyme can also transfer electrons from ubiquinol “upstream” for NAD^+^ reduction (so-called reverse electron transfer (RET)). Under physiological conditions, complex I can catalyze the production of reactive oxygen species (ROS) such as superoxide and hydrogen peroxide and can also be a target of ROS [14]. Another interesting feature of mitochondrial complex I from mammals is the so-called active/deactive (A/D) transition [13], [15], [16]. The existence of two distinct catalytic forms of the enzyme was shown *in vitro* [15], [17], [18], in cultured cells, and in mouse and rat tissue under ischemic conditions [19], [20], [21], [22]. Recently, the structural differences between the two catalytic forms as well as the physiological role of this process were studied in detail by our group and by others. In this review, we aim to summarize current knowledge on the characteristics and mechanism of the A/D transition of mitochondrial complex I. In addition, this review discusses the impact of the A/D transition on the production of ROS by complex I since the modulation of transient “deactivation” of complex I might be a promising strategy to prevent oxidative damage under pathophysiological situations involving tissue ischemia.

## 2 Characterization of transition

**Fig. 1:**
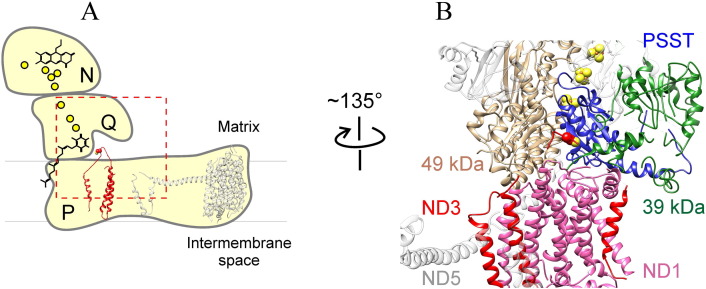
Subunits involved in the A/D transition of mitochondrial complex I. (A) Relative location of the hydrophilic loop of ND3 subunit (THM 1-2^ND3^) based on X-ray structure of *Y. lipolytica* enzyme [1] (PDB ID: 4WZ7). N, Q, and P stand for NADH-dehydrogenase, Quinone reduction, and Proton translocation modules, respectively. The relative positions of ND3 (red) and ND5 (grey) are shown. Yellow spheres indicate the position of FeS-clusters, the positions of FMN and Q are assigned by respective schematic structural formulas. (B) Interface between central subunits of the Q module and the P module. The subunits are colored individually and labelled with text in the same colors (ND3 red, ND1 pink, PSST blue, 49 kDa beige, 39 kDa green). Horizontal transverse helix of ND5 is shown in grey for orientation. Cys-40 (in *Y. lipolytica* corresponding to Cys-39 in the bovine enzyme) is shown as spheres at the proposed location of the partially resolved loop of ND3 (THM 1-2^ND3^).

The A/D transition, which was first characterized for the mammalian enzyme [15], is also evident in other vertebrates and certain yeasts but has not been detected in prokaryotes (*Rhodobacter capsulatus*, *Paracoccus denitrificans*, or *Escherichia coli*) [23], [24]. During steady-state aerobic respiration, the catalytically active form (A-form) is predominant. It couples the physiological oxidation of NADH by ubiquinone and proton translocation with a rapid reaction rate of 10,000 min^− 1^. If the enzyme is incubated *in vitro* at physiological temperatures or *in situ* when respiration is blocked, e.g., by lack of oxygen (ischemia), the A-form spontaneously converts into the deactive, dormant form (D-form). This form of the enzyme has a different conformation and can potentially be reactivated during slow (~ 1 min^− 1^) catalytic turnover(s) of NADH oxidation by ubiquinone [15], [25], [26]. When tested *in vitro*, the D-form demonstrates a considerable lag-phase during continuous assay of the NADH:ubiquinone oxidoreductase reaction. This lag-phase represents the conformational transition of the D-form into the A-form during slow initial catalytic turnover(s), after which complex I becomes fully active [15], [17], [19]. In any given preparation of complex I (e.g., within submitochondrial particles (SMP), there are two different enzyme forms present as a slowly equilibrating mixture [27], [28]. The equilibrium *in vitro* can be rapidly shifted toward the D-form at physiological temperatures, but the addition of both substrates (NADH and Q) can reactivate the enzyme back into the A-form [28]. The kinetics of the A/D transition and the diagnostic activity assays for the determination of the A/D ratio are covered in several comprehensive reviews [13], [16], [28]. We should stress that many aspects of the conformational changes during the transition (A → D or D → A) have not been comprehensively studied and only a few structural differences between the two enzyme forms have been identified to date. From what we know, the A/D conformational changes affect the Q-module at the junction region between the hydrophilic N-module (where all redox centers are localized) and the membrane proton pumping P-module (Fig. 1A).

### 2.1 Subunits involved

The first characterization of a structural change during the A/D transition of mitochondrial complex I was undertaken by Vinogradov's group [29] and were based on the different sensitivities of the A- and the D-form in bovine heart SMP to SH-reactive reagents observed a long time ago [30]. SMP containing the A-form of the enzyme were treated with *N-*ethylmaleimide (NEM), washed, and labelled with *N*-fluorescein maleimide after deactivation. After crude purification of complex I, and separation of the subunits, specific incorporation of the fluorescence label into an unknown subunit of approximately 15 kDa was observed [29]. This technique was further developed by Galkin *et al.* using doubled SDS-PAGE [31] to unambiguously identify Cys-39 of mitochondrially encoded ND3 as the residue that determines SH sensitivity of the D-form [32] (Fig. 1). The predicted matrix orientation of the long, Cys-39-containing hydrophilic loop (THM 1-2^ND3^) connecting the first and second transmembrane helices [32] was later confirmed by the crystal structure of the prokaryotic enzyme (homologous subunit NuoA in *E.* *coli*) [10]. This loop is an important hot spot for mutations in complex I deficiencies. Single amino acid changes in this domain of ND3 lead to various mitochondrial encephalopathies as well as Leigh syndrome, indicating the significance of this loop in complex I function or regulation [33], [34], [35], [36], [37]. Although Cys-39 of ND3 is highly conserved among the eukaryotes (99% conserved over 108 sequences), its presence does not correlate with the apparent ability of complex I to undergo the A/D transition [38].

Despite the recent availability of partial structures of yeast and mammalian complex I, the exact location of the loop is not well defined, probably indicating it is highly flexible in eukaryotic enzymes [1], [2]. In our initial work, we suggested two scenarios explaining the exposure of this region in the D-form: an intrinsic movement of the ND3 loop or changes in position of adjacent subunits [32]. The recent structural data indicate that the second scenario is highly unlikely and suggest that the flexibility of this ND3 hydrophilic loop enables its exposure to the outside after deactivation (see Supplemental Video 1S with the online version of this article), rendering the sensitivity of the D-form to SH-reagents.

Knowing the nature and approximate location of the critical SH-group, we exploited a range of thiol-specific crosslinking reagents to identify the neighboring subunits. Using a 6.8 Å SH/NH_2_ heterobifunctional cross-linker (*N*-succinimidyl 3-(2-pyridyldithio) propionate), a conformation-specific cross-linked product between the ND3 subunit and the accessory subunit 39 kDa (NDUFA9) was observed exclusively in the D-form of the enzyme [39]. The data suggested that the 39 kDa subunit is located near the ND3 loop and might also be involved in the conformational change. In order to examine this further, we applied lysine-specific fluorescence labelling, and a two-dimensional difference gel electrophoresis approach to confirm that the 39 kDa subunit is indeed more exposed in the D-form, than in the A-form [40]. The close proximity of the ND3 loop and 39 kDa subunit was later proven by the X-ray structure of eukaryotic complex I [1], [2]. However, it is hard to predict what part of the 39 kDa subunit is involved since only three quarters of this subunit was determined in the bovine structure. The 39 kDa subunit is homologous to a nucleotide-binding short-chain dehydrogenase/reductase and bears a tightly bound NADPH molecule that is most likely not involved in the redox reactions during steady-state oxidation of NADH [9], [41], [42]. Despite earlier theoretical indications [43] and predictions of membrane-spanning regions software (TMpred), no association of 39 kDa subunit with the membrane was found [1], [2].

Using a similar fluorescent labelling, we identified that the mitochondrially encoded subunit ND1 is also more exposed in the D-form of the enzyme [40]. As later revealed in the structure of *Yarrowia lipolytica* enzyme [1], not only membrane parts of ND1 and ND3 are in close contact, but the hydrophilic loop of ND3 (THM 1-2^ND3^) is in fact located above the transmembrane helixes of ND1 (Supplemental Video 1S). Therefore, it is not surprising that a movement of the ND3 loop upon the deactivation of the enzyme coincides with the exposure of, as yet, a non-identified part of ND1. The position of Cys-39, adjacent to four acidic residues in the interhelical loop of ND1 (TMH5–6^ND1^), could explain the rather basic p*K*_a_ value of 10.2 determined for this thiol group in the D-form of the bovine enzyme [29].

As shown in Fig. 1B, subunits ND3, ND1, and 39 kDa are located at the junction between the hydrophilic and membrane arms of complex I [1], [2], [3]. Furthermore, 39 kDa flanks the Q-module being adjacent to catalytic PSST subunit and partially encloses the ND3 loop (THM 1-2^ND3^) (Fig. 1B, see also Supplemental Video 1S). It is now known that membrane helixes of ND3 and ND1 form the entrance to the binding pocket for the ubiquinone molecule and that they harbour its hydrophobic tail when the head accepts electrons from cluster N2 [1], [3]. Subunits 49 kDa, TYKY, and PSST, located at the interface between P- and N-modules, are also involved in the formation of the Q-binding site (Fig. 1). Based on structural data provided by Zickermann et al. [1], the D-form of complex I represents a conformation in which the access of the ubiquinone head group to the terminal Fes-cluster N2 is somehow restricted. Our recent results [40], together with structural data [1], [2], [3], support the conclusion that activation (i.e., D → A transition) is due to a concerted conformational rearrangement of ND1, ND3, and 39 kDa subunit. This might result in the formation of a functional ubiquinone-binding site in the A-form that is able to catalyze the physiological reaction. Despite numerous attempts utilizing various amino acid side-specific covalent labels, we were unable to detect any subunits in the A-form that were more exposed than in the D-form (A. Galkin, unpublished data). Since only a small number of subunits are differentially exposed in the D-form [32], [40], it suggests rather discrete changes in shape at the quinone-binding region. Therefore, we believe that the resting D-form is a “relaxed” conformation of that region while activation converges the involved subunits and the A-form corresponds to “tense” conformation of the Q-binding site. Although our hypothesis is highly speculative, it may be helpful for further experimental work to test the proposed mechanism. There are several questions aimed to resolve the mechanism of the A/D transition:

(i) Are additional subunits in that region also involved in the A/D transition? Based on the *Y. lipolytica* structure, PSST middle alpha helix (aa 127–135) and loop β1–β2 of N-terminal β-sheet of 49 kDa subunit closely approach the hydrophilic loop THM 1-2^ND3^. However, currently, no data indicate that the position/exposure of these subunits is affected by the A/D transition. Either this is because respective parts of PSST and 49 kDa are buried inside the tertiary structure and are not accessible for modification, or the conformation of these subunits does not change during the A/D transition.
(ii) What could be the driving force of the activation? Assuming that in the D-form the entrance to the active center is somehow restricted [1], and there is an equilibrium between the A- and the D-form [27], the following scenario can be suggested: a ubiquinone molecule, by virtue of Brownian motion, makes repeated attempts to enter the opening at the membrane part of complex I toward the Q-binding site. If the enzyme is in equilibrium, switching between the A- and the D-states, one of these attempts will coincide with an unrestricted state, which would facilitate the substrate entering the hydrophobic ramp [44], [45]. A slow D → A transition can be related to poorly populated conformational states regulated by internal dynamics of membrane subunits at the opening where ubiquinone is able to access the entrance pathway and potentially accept electrons from N2 making a “priming” redox step. Similar mechanisms are recognized for enzymes with a buried active center [46], [47], [48], and the site of ubiquinone reduction, located 30 Å from the opening at the membrane arm of complex I, serves as a good example [45].
  Evidently, energy released from NADH oxidation by the D-form is used to drive the initial activation step resulting in the conformation change. However, the exact molecular mechanism that drives the transition is not clear. The final activation step is completed when the Q-binding site is enabled to catalyze terminal electron transfer from N2 during steady-state reaction and a ubiquinone molecule is present. Since reduced A-form of the idle enzyme is converted into the D-form with the same rate [15], [19], neither NADH alone nor reduction of terminal cluster N2 itself can initiate the activation. Furthermore, the availability of ubiquinole for oxidized enzyme is also not sufficient for activation [19], [49]. A “priming” redox event should be considered as a main cause of conformational changes leading to formation of the functional Q-site. Therefore, it seems reasonable that formation of an ubisemiquinone species within Q-binding cavity may drive slow conformational change [1] as was suggested more than 25 years ago [15]. This “priming” step can be considered as a slow process partially resembling part of the catalytic cycle (one electron transfer from N2 to ubiquinone); however, it is different from any states that occur during the fast steady-state reaction of the A-form.
(iii) Is there any similarity between the D-form and proposed catalytic states of the enzyme? The resemblance of the A/D transition with the states of the enzyme occurring during catalytic turnover merits special consideration. Despite progress in resolving the complex I structure, the actual mechanism of energy transduction from the Q-binding site toward the proton translocating subunits at the membrane part is not completely understood and several models for the coupling have been suggested [8], [50], [51], [52]. Most likely, energy released at the final redox step of electron transfer from N2 to ubiquinone drives the translocation of protons by the mitochondrially encoded subunits in the membrane. A possible connection between a conformation occurring during the final stages of catalytic cycle of complex I (N2 to ubiquinone electron transfer) and the structure of the D-form is not clear. In our opinion, a time point when a ubisemiquinone molecule has been already produced and subunits change from the D to the A-state has been initiated, while the Q-pocket is not yet in a “catalytic state” would correspond to the E-state proposed in Brandt's two-state stabilization-change mechanism [8]. However, during this step, the free energy released in NADH:Q reductase reaction is not transmitted downstream to the P-modules but is instead utilized for maintaining ND3, ND1, 39 kDa, and possibly 49 kDa/PSST subunits together into functional, “tense” Q-pocket capable of rapid catalytic turnover.

### 2.2 Regulation of the A/D transition

In the last 25 years, significant progress has been made in studying of the regulation of the A/D transition, and a number of physiology-relevant effectors have been identified for the mammalian enzyme. Due to some uncertainty in the current literature, we should emphasize the necessity to distinguish between three fundamentally different ways of affecting the equilibrium between the A and the D form *in vitro* and *in situ*. (i) Reversible effects on kinetics of the deactivation/activation (e.g., rate of the change): several low and high molecular weight ligands may influence the kinetics of the A/D transition. (ii) Substrates availability: in steady state in the presence of limited amounts of NADH and ubiquinone, slow flux of electrons via complex I could maintain the enzyme partially in the A-state, shifting A ↔ D equilibrium to the left. (iii) Effect of irreversible covalent modifications of the D-form that prevent reactivation: the persistent inhibition of complex I by nitric oxide (NO) observed in [53], [54], [55] was in fact due to nitrosation of Cys-39 in the D-form by NO metabolites [56]. *In vitro* treatment of such modified enzyme by reducing agents (glutathione or dithiothreitol) could reduce the critical thiol group and fully restore physiological activity. The lifetime of Cys-39 S-nitrosation *in situ* and the nature of enzymatic systems able to reverse covalent thiol modification are still obscure [22], [57]. The effect of deactivation on various covalent modifications of the D-form is covered by Babot et al. [16].

After prolonged incubation of the idle enzyme preparation at different temperatures, only 10% of the enzyme stays in the A-form [27]. Temperature significantly decreases the time to reach equilibrium, so that time necessary to complete the process is in the range of 5–10 min at 35 °C and as long as 60 min at 30 °C (bovine heart SMP *in vitro*). This is correct only for the idle enzyme in the absence of reduced nucleotides and ubiquinone. In conditions when complex I turnover is allowed *in vitro* (e.g., in the presence of oxygen and NADH), enzyme in SMP could be maintained in the A-form for a very long time [56]. This is also true for intact mitochondria, which retain matrix nucleotides: incubation of such preparation aerobically at elevated temperatures unlikely results in complete deactivation due to the slow electron transfer from various endogenous substrates to oxygen via complex I. In practice, conditions providing any slow reaction involving complex I can be used for shifting A ↔ D equilibrium to the left. Therefore, several approaches can be used to support the enzyme in the A-form at 25–35 °C: slow-oxidizing substrate NADPH [29], substoichiometric amount of NADH provided by the regenerating system of alcohol dehydrogenase/ethanol [56], or NADH:fumarate reductase [19], [20].

Provision of the enzyme turnover is opposite to the situation when insufficient oxygen supply is provided *in situ*. In conditions of tissue ischemia, mitochondrial redox centers are over-reduced due to the slowing of cytochrome *c* oxidase. Consequently, complex I turnover becomes restricted by the lack of electron acceptor ubiquinone. Accumulation of reduced ubiquinone not only decreases the availability of substrate for the enzyme but also inhibits the physiological oxidoreductase activity of complex I [58], [59]. In this situation, the steady-state equilibrium in the A ↔ D reaction is shifted to the right and within minutes complex I is converted into the D-form [21], [60]. A similar effect can be induced by metabolic hypoxia [61], a situation when available oxygen cannot be used due to an increase in NO that competes with oxygen and inhibits cytochrome *c* oxidase [61]. More generally, the inhibition of energy transfer downstream of complex I *in situ* (ROS/RNS-damage, release of cytochrome *c* as well as “chemical hypoxia” by cyanide or carbon monoxide) would strongly synergize with hypoxia to induce the deactivation of complex I.

Several physiological low molecular weight effectors influence the kinetics of the A/D transition. Divalent cations and alkalinisation strongly inhibit the activation of enzymes from bovine [62], [63] and rat heart [64] *in vitro*. Recently, we analysed the effects of monovalent cations on the rate of activation. At neutral pH (7.0–7.5), only sodium was able to increase the rate of activation (D → A conversion) while all other alkali cations were not. This stimulating effect of sodium was caused not by an increase in ionic strength but probably by specific effects on membrane subunits. At alkaline pH, all tested metal ions showed a pronounced inhibitory effect on activation, which could be explained by an increase in ionic strength.

It has been shown over the time that lipophilic compounds such as free fatty acids inhibit electron transport from NADH to oxygen via complex I [65], [66]. Recently, it was found that palmitate affects not only physiological NADH:Q reductase activity of complex I, but it also decreases the rate of activation by several orders of magnitude [25]. These intriguing effects of free fatty acids [25], [63] are highly relevant in several physiological situations. Free fatty acid content of the mitochondrial membrane increases several fold in acute cerebral or cardiac ischemia [67], [68], [69]. In ischemic cardiac tissue, the deactivation of complex I [19] occurs in the same time scale as the inhibition of β-oxidation and accumulation of acyl-carnitines and acyl-CoAs [70]. Therefore, we expect that the A/D equilibrium *in situ* could be significantly modulated by the content of fatty acid metabolites that accumulate when the oxygen supply is restricted, and that the rate of deactivation in tissues is probably higher than *in vitro*. The time course of complex I A → D conversion after cardiac arrest showed a faster rate in brain when compared to heart tissue [38]. Since no tissue-specific isoforms of complex I have been characterized so far, the diverse fatty acid and lipid content of brain and cardiac mitochondria may determine differential time frames of the A/D transition [71].

The existence of specific high-molecular weight effectors of the A/D transition has not been studied yet. In principle, proteins from matrix or intermembrane side may interact with complex I and affect the kinetics of the A/D transition. Also, some of the numerous accessory subunits of complex I could regulate the A/D transition. A suppressing effect of methylation-controlled J protein on complex I activity observed in [72] suggests that this protein might regulate the balance between the two forms of the enzyme. Based on *Y. lipolytica* structure, subunits NB4M and ACPM1 (B14 and SDAP orthologs in bovine) form a domain at the base of the hydrophilic arm above the critical ND3 loop. It has been suggested that NB4M could come in contact with the loop and may bind so-far-unidentified factors that can regulate the A/D transition [73].

Another important question is whether any chemical/pharmacological compound could affect activation/deactivation of the enzyme in a turnover-independent way. Rotenone is a classical hydrophobic inhibitor of complex I that binds to the Q-pocket. It was shown that this inhibitor binds to the A-form of the enzyme with an almost two fold higher affinity than to the D-form [27]. Due to this fact, rotenone had a profound effect on the slow equilibration between the A- and the D-form. Incubation with inhibitor partially protected the enzyme against deactivation and reactivated the D-form by shifting equilibrium of the A ↔ D reaction to the left. It is possible that a bound rotenone molecule could act as a clamp, catching the enzyme in the A-conformation [27], or induce activating conformational D → A changes without a turnover.

Pharmaceutical compounds that modulate the A/D transition rate can be of specific therapeutic interest. For a long time, metformin was the first-line drug for the treatment of type II diabetes. It has been reported that metformin, as well as some other biguanides, directly inhibits complex I [74], [75], [76]. Moreover, the rate of the inhibition of NADH oxidation was significantly higher if metformin was preincubated with the D-form than with the A-form [76]. It was suggested that the deactivation of the enzyme may facilitate the binding of biguanides to complex I in the region of the critical ND3 loop (THM 1-2^ND3^) [76]. Analysis of direct effects of biguanides on both forms of complex I revealed that deactivation greatly enhances the sensitivity of the enzyme toward biguanides, leading to the inhibition of the activity [64]. Based on the data presented in both studies, an alternative explanation is also possible. Apparent suppression of NADH-oxidase activity observed in refs. [64], [76] cannot be explained only by the inhibition of the D-form but rather by specific effects of biguanides on the *rate* of activation. Similarly, at alkaline pH (> 8.5) and in the presence of divalent cations (1–5 mM Ca^2 +^ or Mg^2 +^), the rate of the D → A transition is decreased by several orders of magnitude [62]. Hence, the preset time frame of an NADH-oxidase assay might be not long enough to monitor the complete activation of the enzyme (see also diagnostic tests for the A/D ratio determination [16]). When activation is greatly slowed, the rate of NADH oxidation corresponds to the (small) fraction of the A-form that is present during the initial phase of the assay.

## 3 ROS production by complex I and the A/D transition

The mitochondrial complex I has been recognized for decades as one of the main sources of reactive oxygen species inside mitochondria – largely superoxide (O_2_^.−^) and its dismutation product hydrogen peroxide (H_2_O_2_) [77], [78], [79], [80], [81], [82], [83], [84], [85], [86]. Complex I-related ROS production has been directly linked to neurodegenerative diseases [87], [88], oxidative damage occurring during ischemia/reperfusion (I/R) [89], and aging [90], [91]. Complex I is not only a source of detrimental ROS but also a target of oxidative and nitrosative damage in several pathological situations [14], [21], [53], [54], [88], [92]. We identified that sensitivity of complex I to redox modifications is different for the A- and D-forms [14], [16], [21], [22], [56], which makes the A/D transition a key factor in determining enzyme vulnerability during oxidative stress. This part of the review attempts to give an overview of the mechanisms and circumstances that lead to complex I-related superoxide/H_2_O_2_ production and focuses specifically on the impact of the A/D transition and thiol redox modifications in I/R.

### 3.1 ROS generation by complex I

Several factors should be taken into consideration when interpreting experimental data on complex I ROS production. In SMP, complex I redox centers are exposed to the outside medium and in intact mitochondria, the hydrophilic part of the enzyme is in its natural environment surrounded by a highly concentrated solution of proteins and low-molecular weight metabolites. While measuring complex I superoxide production is straightforward in SMP, it is technically challenging, if not impossible, in preparations of intact mitochondria given the presence of numerous ROS-metabolizing systems. In our opinion, data obtained from complex I-mediated ROS generation in SMP and “emission” of ROS [93] in preparations of intact mitochondria should be compared with great caution.

Moreover, recent detailed studies in intact mitochondria revealed that other enzymes (e.g., the dihydroliponamide dehydrogenase subunits of PDH and OGDH complexes) are the major sources of superoxide/H_2_O_2_ in the presence of NADH-generating substrates and inhibitors of the quinone site [86], [94], [95]. This can be explained by the link between these enzymes and complex I via mitochondrial NADH/NAD^+^ pool, so that the inhibition of complex I impedes the reoxidation of NADH, which in turn promotes ROS generation from the dihydrolipoamide dehydrogenases. This finding also has important implications for the oxidative damage that occurs in mitochondria during the inhibition of complex I operating in the forward mode. Complex I generated superoxide is completely released into the mitochondrial matrix [96], [97], while the Q_o_-site of cytochrome *bc*_1_ complex, another mitochondrial superoxide producer [98], releases superoxide mostly into the intermembrane space (IMS) [96], [97]. In accordance with these results, it was recently shown that complex I and complex III ROS production target different protein-thiols in isolated intact rat heart mitochondria, i.e., complex I linked ROS – induced by 2-n-decyl-quinazolin-4-yl-amine (DQA) – only oxidized matrix and inner membrane proteins, while complex III linked ROS – induced by antimycin A – also oxidized proteins of the IMS and the outer membrane [99]. Subunits of the PDH and OGDH complexes were among the targets that have been originally assigned to ROS generated at complex I [99]. Considering the results of Brand and colleagues, these two enzymes are the main ROS producers when complex I is inhibited at the Q-site [86], [94]. Therefore, it seems much more likely that the oxidizing ROS were generated by the intrinsic dihydroliponamide dehydrogenase of those complexes than by the flavin site of complex I. This might also apply to other proteins that have been initially assigned as “complex I ROS targets” [99].

Inhibitory analysis is still the most common tool for the studying production of ROS by mitochondria. Hydrophobic inhibitors like rotenone, piercidin A, or DQA bind at the Q-pocket and do not cause a noteworthy increase in the rate of superoxide production by purified complex I or bovine SMP [81], [82]. However, the fraction of electrons that are transferred onto molecular oxygen is largely increased in the presence of a quinone-like inhibitor (or in the absence of oxidized ubiquinone) [83]. These results seem to be in stark contrast to investigations on isolated mitochondria where hydrophobic inhibitors always increase the rate of ROS emission, indicating that there could be other enzymes involved in H_2_O_2_ formation [80], [94], [100]. An alternative explanation is that in intact mitochondria, the redox state of the NADH pool in the steady state (i.e., in the absence of an inhibitor), is more oxidized than in related experiments with the isolated enzyme or with SMP, where usually 100% NADH is applied for the “control”. Hence, the large increase in intact mitochondria can be – at least partially – explained by a rise of the NADH/NAD^+^ ratio, which largely affects the ROS production by complex I (see below). The situation is complicated by the fact that either superoxide or hydrogen peroxide (H_2_O_2_) could be the primary reactive species produced by complex I. Of the total ROS production, lipid-activated purified bovine heart complex I releases 95% as superoxide [83], whereas the enzyme isolated by Hatefi procedure produces around 80% [101].

**Fig. 2:**
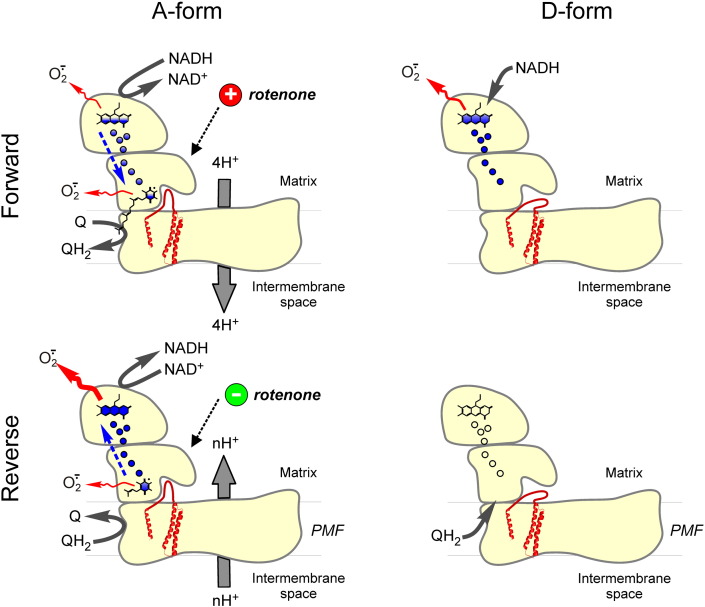
Superoxide generation by complex I is influenced by the direction of electron flow and the A/D transition. Under normal physiological conditions, electrons are transferred from NADH to the Q-pool and superoxide production is low (left, top). Inhibition of complex I by Q-site inhibitors results in an increased superoxide production. Also A → D transition should stimulate superoxide production from the flavin-site (right, top). In the RET mode, proton-motive force (PMF) drives electrons from ubiquinol “uphill” into complex I. The main source under these conditions is still under debate and may include a contribution of the flavin site and the Q-site of complex I (left, bottom). When complex I is in the D-form, RET-driven superoxide production is completely suppressed, because a conformational change in the nearby Q-binding pocket prevents the electron transfer to N2 (right, bottom). FMN, FeS clusters, and ubiquinone molecules are shown schematically. Blue filling of the redox centers of complex I reflects degree of their reduction.

Despite experimental data on purified enzyme, SMP, and intact mitochondria, there is still no absolute consensus in the field on the site and mechanism for ROS production by the enzyme (see the articles by Hirst and Vinogradov in this Special Issue). Complex I can operate in forward and reverse mode as a fully reversible proton pump [8], [102] (Fig. 2, left, bottom). Under physiological conditions, mitochondrial complex I catalyzes the “forward” (i.e., energetically favored “downhill”) electron transfer from NADH provided by matrix substrate dehydrogenases via a chain of iron–sulfur clusters to a ubiquinone (Fig. 2, left, top). This is coupled with translocation of protons from matrix to the intermembrane space. In membrane preparations of the enzyme (SMP or intact mitochondria), it is possible to reverse this reaction (RET). Electrons can be transferred (energetically “uphill” against the difference of redox potentials) from ubiquinol via the FeS clusters to NAD^+^ and most likely proton movement from intermembrane space to matrix drives this process. RET requires the presence of proton-motive force across the membrane and availability of ubiquinol (Fig. 2, left, bottom). The latter can be produced during oxidation of succinate, 3-glycerophosphate, or acyl-CoAs. It has been recognized that ROS production can occur under both operation modes, albeit with different rates [80], [81], [82], [83], [103], [104], [105] and probably also involving different sites [85], [86], [106], [107]. Experiments on isolated enzyme [81], [83], [107] showed that at ambient oxygen concentration the rate of NADH-dependent superoxide production is around 10–40 min^− 1^ (*Y. lipolytica* and bovine complex I) [81], [83]. Although experimental conditions were different, these values correspond to rate obtained from the enzyme in bovine SMP [82], [108].

What could be sources for ROS production in complex I? Thermodynamically, any of the eight FeS clusters of the enzyme may react with molecular oxygen, and the most obvious candidates are low potential cluster N1a [100] and high-potential terminal cluster N2 [101], [109]. However, both FeS clusters are located at great distance from the nucleotide-binding site, and the inhibitory effect of NAD(P)H on superoxide production of complex I is then hard to explain [82]. Mutations in *Y. lipolytica* and *E. coli* affecting redox potential of cluster N1a had no effect on superoxide production [108], therefore excluding it from the list of possible candidates. Redox midpoint potential of N2 is pH-dependent in the range between pH 5.5 and 7.5 [110], [111], which is too low to explain the pH profile of superoxide production of the isolated enzyme (steep increase with alkalinization above pH 7) [81]. As it was shown later, a decrease in the reduction state of N2 was associated with an elevated superoxide production and an increased signal from tightly bound semiquinones [84]. Finally, mutant complex I from *Y. lipolytica* lacking a detectable cluster N2 exhibited the same rate of ROS production as a wild type enzyme [81]. Thus, it provides further evidence for excluding N2 as the site of ROS production.

Tightly bound ubisemiquinones were proposed as a source of ROS in complex I by several groups [80], [84], [112]. Since semiquinones cannot be detected in idle enzyme [113], these intermediates of electron transfer should only be considered in line with a final step of ubiquinone reduction during complex I steady-state activity. However, RET-induced superoxide production by bovine enzyme has a potential dependence consistent with a reduced flavin and not with a ubisemiquinone [107]. Studies with SMP and purified mitochondrial complex I from different sources have revealed that the ratio of NADH/NAD^+^ determines the rate of ROS in the forward mode, i.e., it is highest when [NADH] > [NAD^+^] [82], [83], [101], [114]. Consistent with these results, a similar relation between NAD(P)H oxidation state and superoxide/H_2_O_2_ production has been observed in intact mitochondria [100], [114], [115]. The membrane potential only has a minor effect on the initial rate of ROS production by complex I operating in the forward mode [81], [83], [107], [116]. It should be noted that a steep rise in ROS emission in intact mitochondria with augmentation of potential is most likely due to the increase of reduction state of enzymes redox centers as well as both NADH/NAD^+^ and QH_2_/Q ratio [117].

Most of the experimental data support the view that the reduced FMN is the source for superoxide/H_2_O_2_ in complex I operating in the forward mode [81], [82], [83], [85], [101], [102], [106], [107]. Strong pH dependence of the superoxide production rate and inhibitory effect of NAD^+^ [83] or NADH [82] also indicate that flavin, in its reduced or semi-reduced form, generates superoxide. Dramatic effect of point mutations within the NADH-binding site on ROS production by bacterial enzyme also favors FMN as main site of oxygen reduction by complex I [118], [119]. The rate of superoxide production in the forward mode is highest at NADH concentrations below physiological level in the matrix [82], [101], [120], which could be explained by the blocking of oxygen access to the flavin by bound substrate nucleotides in the active site. The resolved structure of complex I with bound nucleotide [121] suggests that NAD^+^ partially overlays the isoalloxazine ring of the FMN molecule that protrudes into the cavity therefore hindering oxygen access.

Complex I reverse reaction is associated with higher rates of superoxide generation [103], [104], [106], [122], [123], [124]. The uncoupling [82], [104], [117] or inhibition of complex II (reviewed in [125]) – which impedes the build-up of proton-motive force or the reduction of the Q-pool – attenuates the superoxide/H_2_O_2_ generation during RET. Rotenone-like inhibitors acting at the Q-binding site also decrease the ROS production in RET [82], [104], [106], while the same inhibitors increases ROS production in the presence of NADH-linked substrates (Fig. 2, left, top). Diphenyleneiodonium covalently binds to the reduced FMN [126] and also inhibits RET-induced ROS generation in intact mitochondria in the presence of succinate [122], [127], [128]. A detailed analysis by Pryde and Hirst [107] showed that both hydrophobic inhibitors of the quinone site and inhibitors of the flavin site (rotenone/piericidin and ADP ribose, respectively) suppressed RET-driven superoxide production by coupled bovine heart SMP. Hydrophobic, rotenone-like inhibitors decrease the superoxide production from both flavin and quinone sites by interrupting the electron flow from the Q-pool to FMN. On the other hand, flavin site inhibitors a priori only effect ROS production from the flavin site. In addition, binding of NAD^+^ and acetyl-NAD^+^ decreased the rate of succinate-supported superoxide production in coupled SMP [82], [105]. These data favor reduced flavin as the dominant source of ROS during reverse reaction.

A critical issue of these investigations is the fact that the maximal rate of superoxide production by complex I in intact mitochondria [106], [107], [114] is considerably higher than in coupled SMP [107]. In mitochondria, rates during RET are considerably higher than in the presence of NADH-generating substrates and inhibitors of the quinone site. We should stress that when SMP or reconstituted enzyme are used at RET-like conditions in the presence of the proton-motive force and quinole, there is no actual transfer of electrons without exogenously added NAD^+^. In such conditions, the over-reduction of complex I redox centers (including FMN) could result in the generation of ROS. The situation is the opposite in intact mitochondria, where internal NADH/NAD^+^ is always present and therefore affects complex I ROS generation [82], [83], [114]. In investigations with intact mitochondria, it was shown that the superoxide production from the flavin site correlates with the redox state of the NADH/NAD^+^ pool [86], [129]. Applying this to succinate-supported respiration by mitochondria, only ~ 7% of the superoxide/H_2_O_2_ production could be attributed to the flavin site of complex I [86]. This would suggest the possibility that the majority of the rotenone-sensitive superoxide production may come from other sites.

### 3.2 Effects of the A/D transition and redox modifications on superoxide production by complex I and ischemia/reperfusion (I/R) injury

Most experimental data on complex I-related production of ROS by the A and the D-forms could be explained in the view of the recently published structure of mitochondrial enzyme [1], [2]. The functional similarity (i.e., breach in electron transfer between cluster N2 and ubiquinone molecule) between the D-form of complex I and the rotenone-inhibited enzyme can be deduced from the structural data provided by Zickermann et al. [1]. As explained above, the D-form of complex I represents a conformation in which the access of the ubiquinone head group to the terminal FeS-cluster N2 is somehow restricted due to the structural rearrangement of subunits that built the ubiquinone-binding pocket. The functional outcome of this is similar to the blockage of electron transfer within the A-form by a molecule of rotenone-like inhibitor bound at the quinone site. If electrons are supplied in the forward mode from NADH, both scenarios will hamper the terminal electron transfer onto ubiquinone, which in turn leads to an over-reduction of the upstream redox centers and largely increases the chance of ROS production at flavin site. During RET, both scenarios prevent the electron transfer from ubiquinol onto FeS-cluster N2 and thereby superoxide production (Fig. 2, right, bottom), irrespective of whether the reactive species are formed at the quinone or flavin site of complex I. These structural presumptions explain available experimental data very well.

The effect of the A/D transition on the superoxide production by complex I was first investigated by Vinogradov's group [105] using coupled SMP from bovine heart mitochondria. Observed catalytic activities and EPR spectra of the D-form were found to be similar to those of the rotenone-inhibited complex I [105], [130]. The deactivation of the enzyme, as well as binding of rotenone-like inhibitors, has strong effects on both forward/reverse reaction [15] and superoxide production [82], [105].

The inhibition of NADH oxidation by rotenone or locking complex I in the D-form increased the amounts of produced superoxide by around 50% in bovine SMP [82], [105]. A similar increase in superoxide production during NADH oxidation was measured for the D-form using rat heart SMP [64]. This effect was significantly more pronounced at the alkaline pH [64] probably for two reasons. First, after a given period of NADH oxidation, there would be more D-form in the assay since activation (D → A transition) takes much longer at alkaline than at neutral pH [62]. Second, the rate of superoxide production increases steeply with alkalinisation of the assay medium above pH 7.0 [79], [81].

When electrons were supplied from succinate by RET, locking complex I in the D-form almost completely stopped superoxide production [62], [105] (Fig. 2, right, bottom). In the D-form, transfer of electrons from ubiquinol to cluster N2 is interrupted. Therefore, upstream components, including flavin, cannot be reduced, which also impairs ROS production. These fundamental findings provide the mechanistic basis for understanding the critical role that complex I has in generating ROS in early stage of reperfusion.

**Fig. 3:**
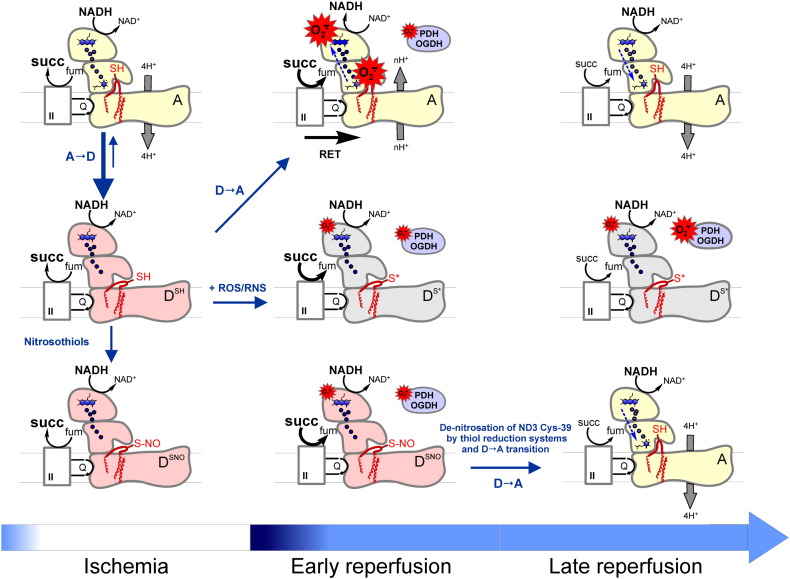
Effect of the A/D transition and differential SH-redox modification on ROS generation during ischemia/reperfusion. Under normoxic conditions, the large majority of complex I is in the A-form, and therefore, Cys-39 of ND3 is not accessible (top panel). During ischemia, A → D transition takes place in a time-dependent manner and Cys-39 is exposed (D^SH^) (left, middle). The matrix succinate concentration is highly increased by the reversal of complex II. In the early phase of reperfusion, this succinate can be oxidized and this drives RET if complex I is in the A-form (center, top). This is accompanied by a massive oxidative burst via complex I. Complex I in the D-form is only marginally contributing to this burst at the flavin site fueled by electrons from NADH. Exposed Cys-39 is now prone to oxidation by ROS or RNS, leading to an irreversibly deactivated complex (D^S⁎^) (center, middle). While some of the A-form complex I that has not been damaged in the burst phase could go back to normal catalytic function after oxygen has been reintroduced, the D^S⁎^ complex will directly and indirectly (via NADH-linked matrix substrate dehydrogenases, e.g., OGDH and PDH) contribute to an increase in ROS generation (center, middle). In the presence of nitrosothiols (bottom panel), the modification of Cys-39 locks complex I in the D-form (D^SNO^) (left, bottom), which reduces the damaging oxidative burst upon reperfusion (center, bottom). However, unlike the oxidized D^S⁎^-form, the D^SNO^ can be recovered by the action of thiol reducing systems and then undergo the D → A transition (right, bottom). OGDH and PDH stand for 2-oxoglutarate dehydrogenase and pyruvate dehydrogenase, respectively; II stands for respiratory complex II (succinate dehydrogenase).

Increased ROS production by mitochondria is associated with detrimental consequences in several pathophysiological situations including I/R injury [131], [132], [133]. Decreasing oxidative stress during the early stage of reperfusion by various means (preconditioning or antioxidant therapy) protects tissues from injury [14], [132], [134], [135]. At the same time, acute ischemia induces the deactivation of complex I within minutes in tissues like heart, brain, muscles, and kidney [19], [21], [22], [60]. If complex I deactivation occurs during the ischemic period, what would be the adaptive role of this transition? A detailed explanation is shown in Fig. 3. Paradoxically, complex I deactivation results in an apparent increase in the rate of superoxide production rates in forward reaction (Fig. 3, center, middle; see also Fig. 2, right, top). According to mass action law, the reaction rate is determined by reactant concentration. For the superoxide production, one of the reactants is the reduced form of complex I ROS-generating site (e.g., flavin) and the second is oxygen. If oxygen is present, the rate of the superoxide production by the D-form is greater than by the A-form due to the higher degree of flavin reduction. At the same time, in conditions of ischemia, deactivation as such does not make any effect on complex I ROS generation, which is negligible when [O_2_] = 0 (Fig. 3, left column). A completely different situation occurs after ischemia at the early stage of reperfusion, when oxygen is introduced to the system where all redox centers are reduced and a potential across the membrane is present (Fig. 3, center, top). High succinate concentration can accumulate under ischemia/hypoxia in various tissues, [136], [137], [138], [139]. In these conditions, complex I is potentially able to catalyze RET and therefore generates superoxide at a considerable rate [106], [124], [140] (Fig. 3, center, top). The deactivation of the enzyme *in situ* may prevent RET-mediated ROS production leading to oxidative stress and tissue injury at the beginning of reperfusion. After the ischemic period, most of the complex I is present in the D-form and is unable to catalyze electron transfer from ubiquinol to flavin during oxygen resupply. Therefore, the slow activation of the enzyme (D → A transition) upon tissue reoxygenation may function as an intrinsic protective mechanism and decrease ROS production at the level of complex I. Moreover, the deactivation of the enzyme can prevent the burst of respiration via retention of electron transfer from the reduced NADH pool downstream of complex I (i.e., to complex III) at the early reperfusion stage, when the oxygen level is high and metabolic intermediates are not balanced. Therefore, transition of complex I into the D-form in ischemia plays a significant role in attenuating the “oxidative burst” that occurs during I/R [21], [22].

This mechanistic model explains very well the experimental data showing that the reversible inhibition of complex I during cardiac or cerebral postischemic reperfusion would protect mitochondria and decrease I/R damage in various animal models [22], [138], [141], [142], [143], [144]. At the same time, deactivation increases susceptibility of complex I to covalent modifications such as nitrosation and oxidation. Complex I inhibition by NO metabolites was demonstrated in preparations of intact mitochondria and cells a long time ago [145], [146], [147]. Prolonged exposure to high concentrations of NO led to the persistent inhibition of complex I in cells, which was attributed to nitrosation of critical thiol residue(s) [53], [54], [55], [148], [149], [150]. Later, we found that sensitivity of complex I to peroxynitrite and S-nitrosothiols is governed by the A/D status of the enzyme [19], [56]. NO metabolites can react with critical Cys-39 of the ND3 subunit in the D-form and arrest its activation [19], [56] (Fig. 3 middle and bottom rows). It was possible to restore the activity of the S-nitrosated enzyme by reducing agents [19], [56] (Fig. 3, bottom panel), while oxidation of Cys-39 by peroxynitrite or ROS was found to be irreversible (Fig. 3 middle, center) [19], [21], [56]. In a further elegant study from Murphy's laboratory, Cys-39 was identified as a critical residue nitrosated by mitochondrially targeted nitrosothiol MitoSNO which resulted in the inhibition of complex I [22]. Administration of the MitoSNO before reperfusion significantly decreased volume of cardiac infarction and decreased oxidative damage during I/R [22], [151]. It was proposed that nitrosation of Cys-39 delays activation of the enzyme after reintroduction of oxygen and therefore decreases production of ROS. Due to the reversibility of S-nitrosation, the cysteine residue can be subsequently recovered by a denitrosating thiol-reducing system in the mitochondrial matrix [22], [152], [153], [154] (Fig. 3, right, bottom). Importantly, it seems plausible that in conditions of oxidative or nitrosative stress, transient nitrosation (Fig. 3, bottom row) prevents oxidation of critical Cys-39 of ND3 by ROS or peroxynitrite (Fig. 3, center row), protecting the enzyme from irreversible damage. The exact timeframe of such process *in vivo* is not exactly known at present, but it is likely to occur within minutes [22].

**Fig. 4:**
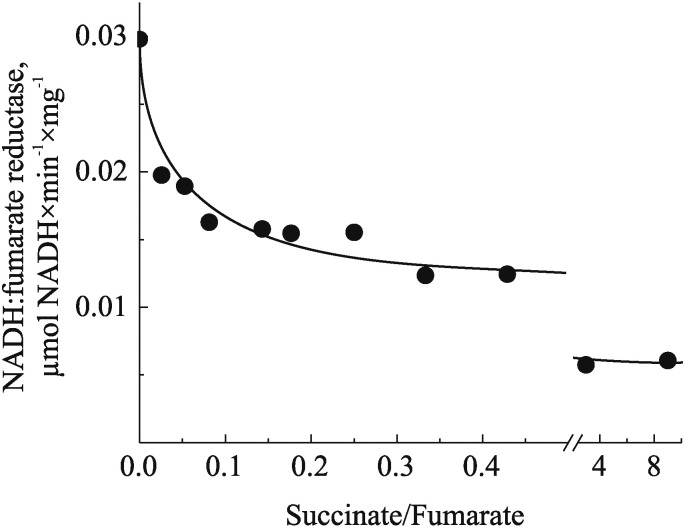
The dependence of NADH:fumarate reductase activity of SMP on succinate/fumarate ratio. Bovine heart SMP were treated as described earlier but without oligomycin incubation [56]. NADH:fumarate reductase was measured spectrophotometrically at 340 nm (*ε*_340_ = 6.22 mM^− 1^ × cm^− 1^) at 25 °C in the standard mixture comprised of 125 mM KCl, 14 mM NaCl, 20 mM HEPES, 0.2 mM EGTA pH 7.2, 2 mM KCN, different succinate/fumarate ratios (total concentration kept 20 mM), and SMP (75 μg/ml). The reaction was initiated by 130 μM NADH followed by addition of succinate. The NADH-oxidase activity of SMP, measured under exactly the same conditions without cyanide, was 2 μmol NADH × min^− 1^ × mg protein^− 1^ and was not affected by the presence of succinate up to 10 mM. All activities were fully sensitive to 1 μM rotenone.

Complex I could contribute to I/R damage via other mechanisms. A detailed metabolomic analysis revealed that ischemic accumulation of succinate observed previously in many laboratories [136], [137], [138], [155] is mediated by the reversal of complex II reaction [139]. Succinate dehydrogenase can reduce fumarate to succinate via reoxidizing ubiquinol, provided by oxidation of NADH by complex I. Reversal of succinate dehydrogenase has been observed many years ago in bovine SMP as the NADH:fumarate reductase reaction is able to support ATP production [156]. On the other hand, the rate of fumarate reduction *in vitro* is only 1–2% of the full NADH-oxidase reaction and it drops dramatically with an increase in the succinate/fumarate ratio (Fig. 4). Therefore, it is hard to explain how this reaction could contribute to the ischemic conversion of fumarate to succinate in the presence of a high concentration of the latter proposed in [139]. One possibility is that *in vivo* succinate generated from fumarate by complex II is readily transported from the matrix to the cytoplasm, so that low succinate/fumarate ratio allows the reversal of the enzyme. At the same time, slow NADH:fumarate reductase reaction was shown to support complex I in the A-form during ischemia [19], [60]; therefore, abating the protective ischemic deactivation of the enzyme considered above. The accurate estimation of the deactivation dynamics, potential fumarate reductase activity *in situ*, and determination of rate and nature of possible covalent modification are a prerequisite for the development of any therapeutic intervention aimed on attenuation of I/R damage. In addition, a role of OGDH should be further reinforced, since this complex is linked via the NADH/NAD^+^ pool affecting complex I-related ROS production. Recent data unambiguously support the view that complex I-related ROS and initial mitochondrial energy failure are largely responsible for the damage that occurs upon reperfusion after a prolonged ischemic period in organs like heart or brain. The ROS inflict severe oxidative injury and can lead to mitochondrial permeability transition, apoptosis, and necrosis determining long term inflammatory tissue response [89], [157].

## 4 Conclusions and perspectives

Impressive progress has been achieved in the last years in studies of the structure and molecular biology of complex I. Vast amounts of data on biomedical implications of this enzyme have also been accumulated. The surprising success of potential complex I inhibitors for averting cognitive decline in Alzheimer's disease requires additional studies for understanding the mechanisms behind its therapeutic actions [158]. Recent phase I clinical trial of the therapeutic compound R118 resulted in a number of serious adverse effects due to complex I inhibition, representing a potential risk to the recipients of mitochondria-targeted drugs [159]. We would like to emphasize that the studies of the regulation of complex I remain an important area for current translational medicine [4], [22], [158], [160], [161].

There are still many open questions in regard to the A/D conformational change. What is the driving force for the A/D transition, and are there any other subunits involved in the conformational change? What is the physiological role of the enzyme deactivation in ischemia and is it possible to affect the A/D ratio *in situ*? Of the greatest importance is the identification of compounds specifically targeting the rate of activation or deactivation, so that we could modulate mitochondrial response to tissue ischemia. We believe that further characterization of the A/D transition may provide an understanding of the regulation of the mitochondrial response to hypoxia and help to develop novel therapeutic interventions for conditions like stroke, cardiac infarction, and other ischemia-associated pathologies.

The following are the supplementary data related to this article.

Supplemental Video 1S Subunits involved into A/D transition of mitochondrial complex I based on *Y. lipolytica* structure (PDB ID: 4WZ7). The protein is shown in grey and subunits involved into A/D transition shown in color using a cartoon representation (ND3 red, ND1 pink, PSST blue, 49 kDa beige, 39 kDa green). Iron–sulfur clusters are represented as yellow spheres. Probable conformational change of partially resolved hydrophilic loop of ND3 THM 1-2ND3 in the A and in the D-form is schematically shown at the end of the clip.

## Transparency document

Transparency document.
