# Supplementary figures and images for: Ischemic A/D transition of mitochondrial complex I and its role in ROS generation
Source: Biochim Biophys Acta. 2016 Jul;1857(7):946–57. doi: 10.1016/j.bbabio.2015.12.013 (PMC4893024; doi:10.1016/j.bbabio.2015.12.013)

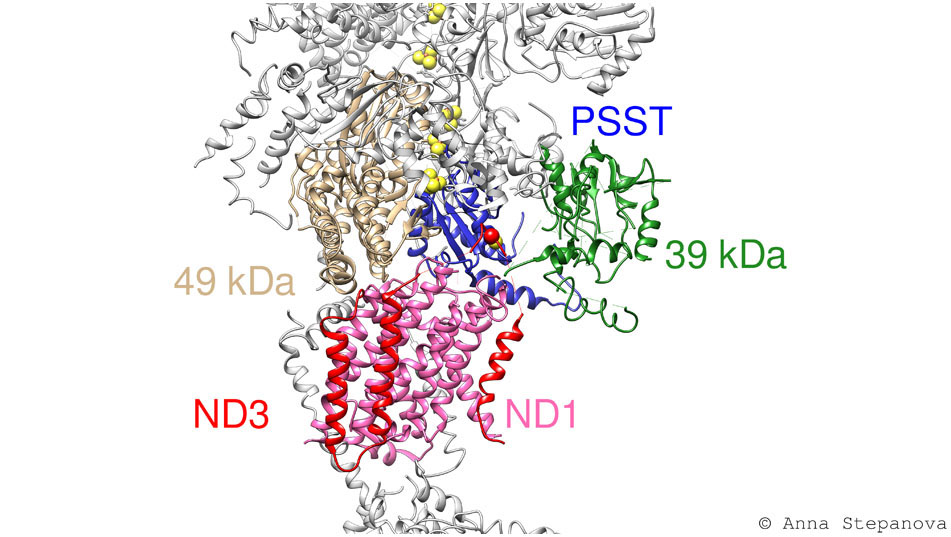

Supplement: Supplemental Video 1S — Subunits involved into A/D transition of mitochondrial complex I based on Y. lipolytica structure (PDB ID: 4WZ7). The protein is shown in grey and subunits involved into A/D transition shown in color using a cartoon representation (ND3 red, ND1 pink, PSST blue, 49 kDa beige, 39 kDa green). Iron–sulfur clusters are represented as yellow spheres. Probable conformational change of partially resolved hydrophilic loop of ND3 THM 1-2ND3 in the A and in the D-form is schematically shown at the end of the clip. [file mmc1.jpg]
